# Influence of HiPIMS Pulse Widths on the Structure and Properties of Copper Films

**DOI:** 10.3390/ma17102342

**Published:** 2024-05-15

**Authors:** Xincheng Liu, Heda Bai, Yongjie Ren, Jin Li, Xiangli Liu

**Affiliations:** 1 School of Materials Science and Engineering, Harbin Institute of Technology (Shenzhen), Shenzhen 518055, China; 22s155058@stu.hit.edu.cn (X.L.); 20b955008@stu.hit.edu.cn (H.B.); 22s155111@stu.hit.edu.cn (Y.R.); 2 Institute of Special Environments Physical Sciences, Harbin Institute of Technology (Shenzhen), Shenzhen 518055, China; jinli2019@hit.edu.cn

**Keywords:** Cu film, HiPIMS, magnetron sputtering, plasma, pulse width

## Abstract

High-power pulse magnetron sputtering is a new type of magnetron sputtering technology that has advantages such as high peak power density and a high ionization rate compared to DC magnetron sputtering. In this paper, we report the effects of different pulse widths on the current waveform and plasma spectrum of target material sputtering, as well as the structure and properties of Cu films prepared under the same sputtering voltage and duty cycle. Extending the pulse width can make the sputtering enter the self-sputtering (SS) stage and improve the ion quantity of sputtered particles. The Cu film prepared by HiPIMS with long pulse width has higher bond strength and lower electrical resistivity compared to the Cu film prepared by short pulse width. In terms of microstructure, the Cu film prepared by HiPIMS with the long pulse width has a larger grain size and lower micro-surface roughness. When the pulse width is bigger than 200 μs, the microstructure of the Cu film changes from granular to branched. This transformation reduces the interface on the Cu film, further reducing the resistivity of the Cu film. Compared to short pulses, long pulse width HiPIMS can obtain higher quality Cu films. This result provides a new process approach for preparing high-quality Cu films using HiPIMS technology.

## 1. Introduction

Magnetron sputtering is a physical vapor deposition (PVD) technique commonly used to prepare various thin film materials such as metals, ceramics, semiconductors, etc. [1]. In the electronic packaging industry, magnetron sputtering is commonly used for the preparation of metal coatings on ceramic substrates [2].

The use of traditional DC magnetron sputtering (DCMS) is often limited by the melting point of the material itself and the cooling system of the equipment, resulting in limited power density that can be applied to the target material [3]. This limits the energy and ionization rate of particles ejected from the target material [4,5]. A low ionization rate will lead to poor acceleration of substrate bias, and lower energy will result in poor quality in the deposited films [6,7,8].

High-power pulse magnetron sputtering (HiPIMS) enables the target material to achieve higher power density by applying a high-power pulse power supply to the magnetron sputtering process [9,10]. The most significant feature of HiPIMS is the ability to generate high-ionization-rate plasma during the sputtering process [11]. High-ionization-rate plasma bombards the substrate under substrate bias, resulting in a thin film with good density and high adhesion between the film and substrate [5,12,13]. At the same time, HiPIMS has the characteristics of high peak power, low pulse frequency, and low duty cycle, which reduces the average power and avoids the phenomenon of target material melting due to high power and insufficient water cooling ability [14,15]. The characteristics of HiPIMS high-density plasma have aroused great interest in related industries. Compared to traditional DCMS, HiPIMS can generate plasma densities of the order of magnitude of 1018–1019 per cubic meter [16,17]. The sputtering material ionization rate can reach 50–90% under certain conditions, and the energy and density of the plasma are very high, as the energy of metal ions can reach 100eV while the energy of inert gas Ar^+^ can also reach 30 eV. HiPIMS can also produce a large number of high-valence gold ions, with an average charge state of ions usually greater than 1 [18,19,20,21].

As is well known, copper is a high-performance material with good conductivity, thermal conductivity, and ductility. Due to its excellent performance, copper thin films are often used as conductive coatings [22], electromagnetic shielding coatings [23], and thermal conductive coatings [24]. Moreover, copper, as a low-cost metal, has a conductivity very close to that of precious metal silver and gold. Therefore, it is also considered the most ideal and promising conductive material for application in microelectronic components, and it is also an ideal conductive layer material for electronic packaging substrates [25,26,27]. The microstructure of copper thin films can affect their physical properties, such as conductivity and mechanical properties [28,29].

Existing research has focused on the effects of peak power and substrate bias of HiPIMS on the structure and properties of film layers, as well as comparing the differences in structure and properties between HiPIMS- and DCMS-prepared films. There is little mention of the regulation of the HiPIMS pulse waveform, such as the influence of pulse width on film layer preparation. In this study, different copper thin films were prepared using different pulse forms, and the effects of different pulse widths on the microstructure and electrical properties of copper thin films were also studied. The current waveforms and the plasma emission spectra were investigated during the sputtering. At the same time, copper thin films were prepared on silicon carbide ceramics commonly used as ceramic substrates for electronic packaging. The effects of different pulse widths and frequencies on the bonding strength of copper thin films on silicon carbide ceramics was studied.

## 2. Materials and Methods

### 2.1. Specimen Preparation

**Figure 1:**
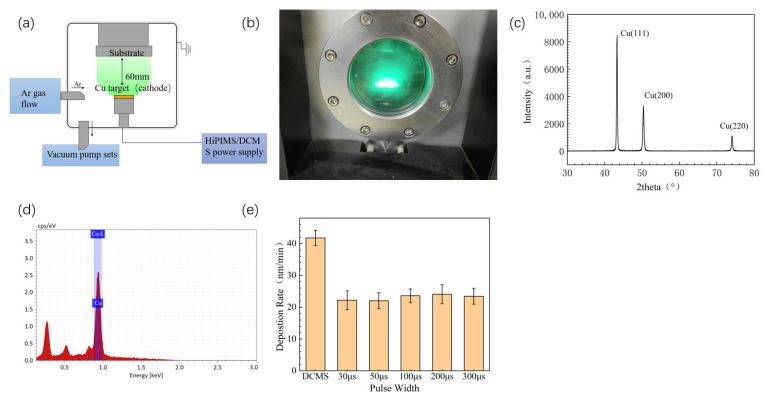
(**a**) The structural diagram of the magnetron sputtering instrument, a single arrow represents the flow direction of Ar and the gas inside the chamber, while a double arrow represents the distance; (**b**) glow during copper sputtering; (**c**) X-ray diffraction of copper target; (**d**) energy-dispersive X-ray spectroscopy of copper target, blue represents the spectral position of copper element; (**e**) deposition rate of different power supply parameters.

The substrate used in the experiment involved 8 inch, 400 μm silicon wafers provided by Zhejiang Lijing Electronics Co., Ltd. (Hangzhou, China). The silicon carbide ceramic was 6 mm × 6 mm × 3 mm, regular hexagon, provided by Fuzhou Kunpeng Optoelectronic Technology Co., Ltd. (Fuzhou, China). Silicon wafers were cut into dimensions of approximately 10 mm × 10 mm using a diamond cutting knife. Before placing the sample in the vacuum chamber, the sample was cleaned with an alcohol bath and an ultrasonic process. The copper target (50.3 mm diameter, 3 mm thickness, 99.99% purity) used in the experiment was provided by Muta New Materials Company (Langfang, China). The X-ray diffraction and energy-dispersive X-ray spectroscopy of copper target are shown in Figure 1c,d. The magnetron sputtering instrument (DTS-200 model) was provided by Pengcheng Semi-conductor (Shenzhen) Co., Ltd. (Shenzhen, China) The structural diagram of the magnetron sputtering instrument is shown in Figure 1a. The chamber was vacuumed continuously by the vacuum pumps for more than 40 min. The volume of the chamber is approximately 0.125 m^3^. The vacuum pumps consist of a mechanical pump (8 L/s model) provided by Ningbo BaoSi Energy Equipment Co., Ltd. (Ningbo, China) and a molecular pump (600 L/S model) provided by Beijing Zhongke Keyi Co., Ltd. (Beijing, China). When the background vacuum was below 3 × 10^−3^ Pa, Ar was introduced and the Ar gas flow was set to 100 sccm. The pressure was adjusted to 0.5 Pa. The purity of Ar was 99.999%, provided by Shenzhen Shente Gas Co., Ltd. (Shenzhen, China). After the pressure was stable, the power was turned on, the target was pre-sputtered by DCMS for 5 min, the average power was 180 W, and then the samples were prepared according the parameters. The sample parameters are shown in Table 1. The deposition rate is shown in Figure 1e. The thickness of the films was around 900 nm.

### 2.2. Structure and Property Characterization

The current waveform of Cu target material during sputtering was measured by an oscilloscope, model TBS1000X, provided by Tech Technology Co., Ltd. (Shanghai, China). The plasma spectrum of the Cu target material during sputtering was measured by a multi-channel spectrometer, model ULS2048CL, provided by Avantes (Eindhoven, The Netherlands). The conductivity of Cu film was measured by a four-probe tester, model RTS-9, provided by Guangzhou Four Probe Technology Co., Ltd. (Guangzhou, China). The copper film was studied using an X-ray diffractometer provided by the Rigaku Corporation (Tokyo, Japen), model Rigaku SmartLab (9 kW), and the residual internal stress of the Cu film was tested. The XRD pattern was measured using a grazing incidence mode, with the grazing incidence angle ω at 2° and a scanning range of 2 θ. The scanning speed was 4°/min, ranging from 30° to 80°. The microstructure of Cu film was studied using environmental scanning electron microscopy, model Gemini SEM 360, provided by Carl Zeiss AG (Oberkochen, Cermany). The shooting voltage was 2 kV and the shooting aperture was 30 μm. Atomic force microscopy was used to study the micro-surface roughness of Cu films via a model of Dimension Icon close loop provided by Bruker Nano Inc. in the Billerica, MA, USA.

**Figure 2:**
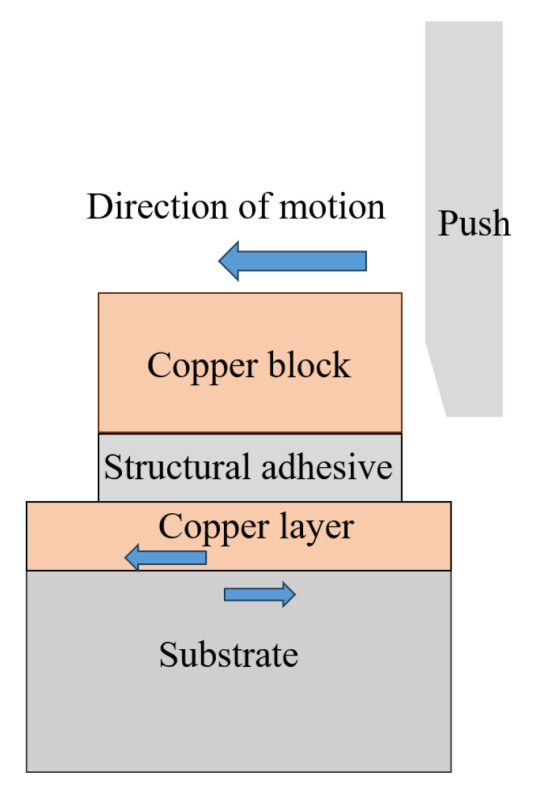
Schematic diagram of shear strength testing method between Cu film and ceramic substrate.

The bonding strength between Cu film and ceramic substrate was measured using a push–pull tester, model Dage4000, provided by Dage Group (Aylesbury, UK). The schematic diagram of the testing method is shown in Figure 2. On one side of the Cu film of the prepared sample, structural adhesive was used to bond the Cu film with the metal block. The copper block was used in this experiment. Subsequently, it was installed on the push–pull tester, and the push blade moved forward to push the metal block. When the thrust reached a certain value, the Cu film was torn off. The shear strength between the Cu film and the ceramic substrate was determined by dividing the reading of the push–pull tester by the area of the torn film.

## 3. Results and Discussion

### 3.1. Target Current and Plasma Spectrum

**Figure 3:**
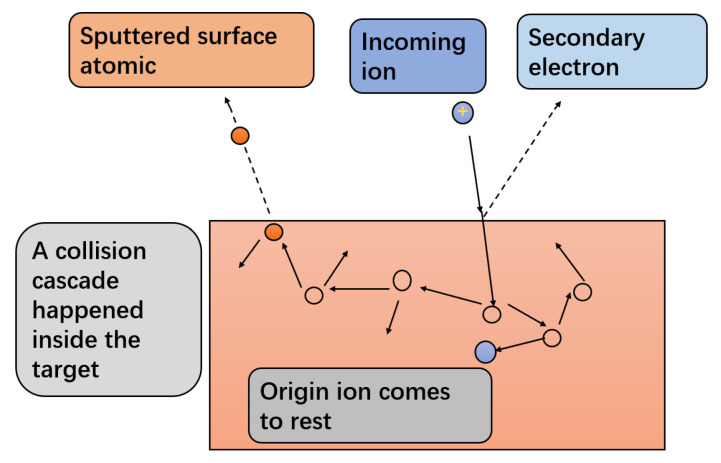
Schematic diagram of a collision cascade in the target.

**Figure 4:**
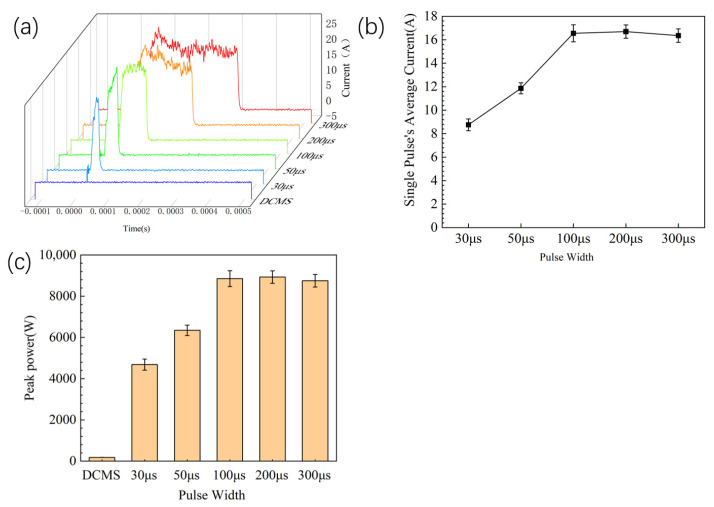
(**a**) Target current waveform diagram; (**b**) the single pulse average current calculated by integrating a single pulse; (**c**) the actual peak power of different power supply parament.

The current waveforms of copper targets with different HiPIMS parameters are shown in Figure 3. The target current is mainly determined by the positive ions (working gas ion Ar^+^, target ion Cu^+^) and secondary electrons that bombard the target material. During the sputtering, the target cathode voltage attracts positive ions to bombard the surface of the target material. A collision cascade is triggered inside the target, caused by an energetic ion, knocking off one or more surface atoms. Further to the surface atoms, secondary electrons may also be emitted [12]. The liberated surface atoms can move to the substrate and condense there, thereby contributing to forming a film. Then, a process of nucleation and growth occurs on the substrate. Figure 3 shows the energetic ions penetrate the target and cause collision cascades in the target. The higher the target current, the more positive ions bombard the target material and the more secondary electrons escape from the surface of the target material, indicating a faster sputtering rate of the target material. Meanwhile, the waveform of the target current can also directly reflect the characteristics of pulse discharge. From Figure 4a, it can be seen that, for samples with a current pulse width of 100 μs or more, the target current rises to the plateau region for stable discharge and the target current waveform shows an approximate square wave shape.

Huo et al. [30] proposed that, in the early stage of target discharge, the target current mainly depends on the working gas Ar^+^. After the target current reaches the first peak, due to the thinning of the gas, the target current decreases and then enters the plateau period. After the target current enters the plateau period, sputtering enters the self-sputtering stage. During the pulse process, the sputtered atoms partially replace the working gas atoms near the target, and in some cases, the sputtered atoms undergo electron shock ionization. A portion of these ions are attracted to the target surface by cathode detachment, and new materials are sputtered onto the target material. This is called self-sputtering (SS) [31,32].

From the current waveform in Figure 4a, it can be seen that the DCMS target current waveform is a line, the current is about 0.5 A. when the pulse width is 200 μs or 300 μs, and the target current passes through the first peak and then decreases into the SS stage. When the pulse width is 100 μs, the target current does not fully enter the SS stage of platform discharge after the first peak. When the pulse width is 30 μs, 50 μs, the target current shows a peak shape, and the discharge stage only stays at the discharge stage that depends on the working gas Ar^+^.

Integrating the HiPIMS pulse waveform to obtain the actual average current of a single pulse, as shown in Figure 4b. The integral calculation formula [33] is

(1) $$Ia=\frac{\int_{0}^{t}I\;dt}{t},$$

When the pulse width is 50 μs or 30 μs, the target material does not discharge smoothly, and the pulse discharge has already ended. Therefore, the average current of a single pulse is much smaller than the average current of a pulse width of 100 μs or 200 μs. Therefore, when the pulse width is 50 μs or 30 μs, the actual sputtering power is much smaller than the pulse width of 100 μs or more. The actual peak power [33] is calculated by

(2) P=U×Ia,

where *U* is the sputtering voltage and *P* is the actual peak power. The actual peak power is shown in Figure 4c. As DCMS does not have a pulse, the peak power of DCMS is its average power.

**Figure 5:**
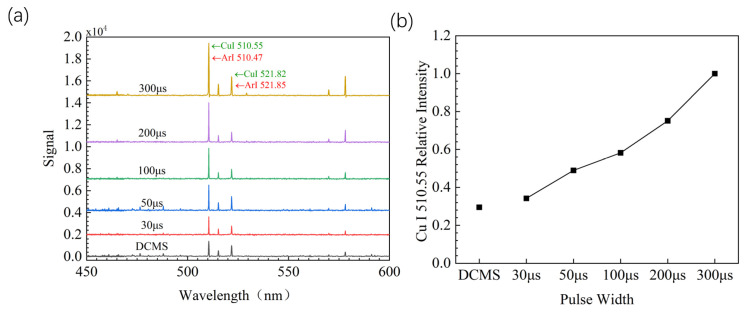
(**a**) Plasma emission spectra with different power supply parameters; (**b**) Cu I 510.55 relative intensity of different power supply parameter.

The plasma emission spectrum is shown in Figure 5a. Positive ions in plasma mainly include Ar^+^ ions and target Cu^+^ ions. The Cu^+^ and Ar^+^ ion signals of HiPIMS are significantly higher than those of DCMS, indicating a higher density of Cu^+^ and Ar^+^ ions. This is obviously due to the fact that the HiPIMS pulse discharge method can achieve higher power density compared to DCMS. As the pulse width of HiPIMS increases, the signals of Cu^+^ ions and Ar^+^ ions increase, indicating an increase in the density of Cu^+^ ions and Ar^+^ ions. Obviously, this is due to the increase in pulse width, and the discharge mode of the target material has shifted from the discharge stage of the working gas Ar^+^ to the discharge stage of SS. Due to entering the SS stage, the plasma concentration increases with the duration of the SS stage, especially for Cu^+^ ion. The Cu I 510.55 relative intensity of the different power supply parameter is shown in Figure 5b, which is calculated by Cu I 510.55 relative intensity = Cu I 510.55 intensity of different parameters/Cu I 510.55 intensity of 300 μs pulse width. The peak in 521.82 nm overall increases with pulse width. There is a decrease at 50 μs to 100 μs. This because the pulse width increased, the power of sputtering increased, and the plasma’s energy increased. More plasmas spectra appear at 510.55 nm, which caused the decrease in peak value of 521.82 nm.

### 3.2. Microstructure and Analysis

**Figure 6:**
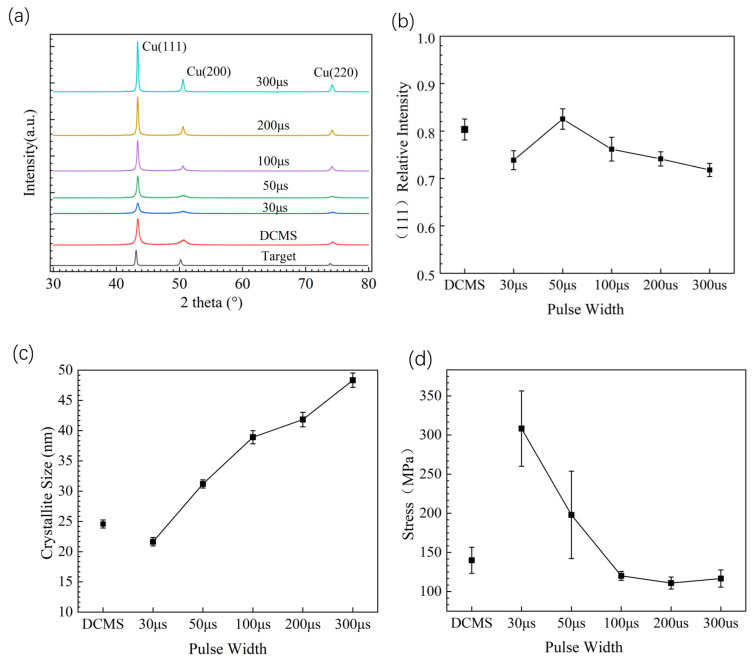
XRD characterization of Cu film with different power supply parameters. (**a**) X-ray diffraction of Cu film with different power supply parameters; (**b**) relative intensity of Cu film (111) crystal planes with different power supply parameters; (**c**) grain size of Cu film with different power supply parameters; (**d**) residual stress of Cu film with different power supply parameters.

The X-ray diffraction patterns of copper films prepared with different parameters are shown in Figure 6a. Overall, the copper film prepared with various parameters exhibits a polycrystalline structure with (111) preferred orientation on the crystal plane. This is because the (111) crystal plane has the lowest surface energy. As the pulse width increases, the XRD diffraction peak of the copper film gradually increases, indicating an increase in the crystallinity of the copper film. The relative strength of copper film (111) crystal planes prepared with different parameters is shown in Figure 6b. The relative strength of (111) crystal planes is calculated from (111) relative intensity = (111) intensity/[(111) intensity + (200) intensity + (220) intensity]. At 30–50 μs, the relative intensity of the (111) crystal plane increases, but as the pulse width continues to increase, the relative intensity of the (111) crystal plane gradually weakens. This indicates that, as the pulse width increases, the orientation of other oriented crystal planes, such as the (200) and (220) crystal planes, increases. For face-centered cubic lattices, the (111) crystal plane is the most stable because it has the lowest surface energy (γ(111) < γ(100) < γ(110)) [34,35]. This is clearly the result of the interaction between surface energy and strain energy.

According to the Scherrer formula, the crystallite size of the copper film prepared with each parameter is estimated as shown in Figure 6c. The Scherrer formula [36] is,

(3) $$D=\frac{K\gamma }{B\;cos\theta },$$

where *D* is the crystallite size, *K* is the Scherrer constant, *B* is the half peak width or integrated width of the diffraction peak of the measured sample, *θ* is the Bragg angle, and *γ* is the wavelength of the X-rays.

Obviously, as the pulse width increases, the crystallite size of the copper film gradually increases. The crystallite size of the copper film prepared with a 300 μs pulse width reaches 48 nm, while the crystallite size of the copper film prepared with a 30 μs pulse width only reaches 21 nm. In a long pulse width discharge state, the ion quantity is higher, and the thin film can obtain more energy to promote crystallite growth. In a short pulse width state, the ion quantity is low, resulting in insufficient energy for crystallite growth.

The internal stress of copper films prepared with different parameters was tested by XRD, as shown in Figure 6d. Due to the difference in thermal expansion coefficient between the metal copper and the substrate, the overall copper film is subjected to tensile stress. According to the previous analysis, when the pulse widths are 30 μs and 50 μs, the actual sputtering power and ion quantity is low, leading to low energy during the preparation of thin films, which can then lead to structural looseness, resulting in increased tensile stress in the films. The increase in energy during film preparation can increase the density of the film, thereby reducing the tensile stress of the film. However, when the film exhibits compressive stress, the increase in energy will increase the compressive stress. Therefore, the obtained copper film exhibits significant tensile stress. When the pulse is greater than 100 μs, the ion quantity is higher, and the sputtered particles obtain more energy. The overall internal stress of the copper film is relatively small, with values below 150 MPa.

**Figure 7:**
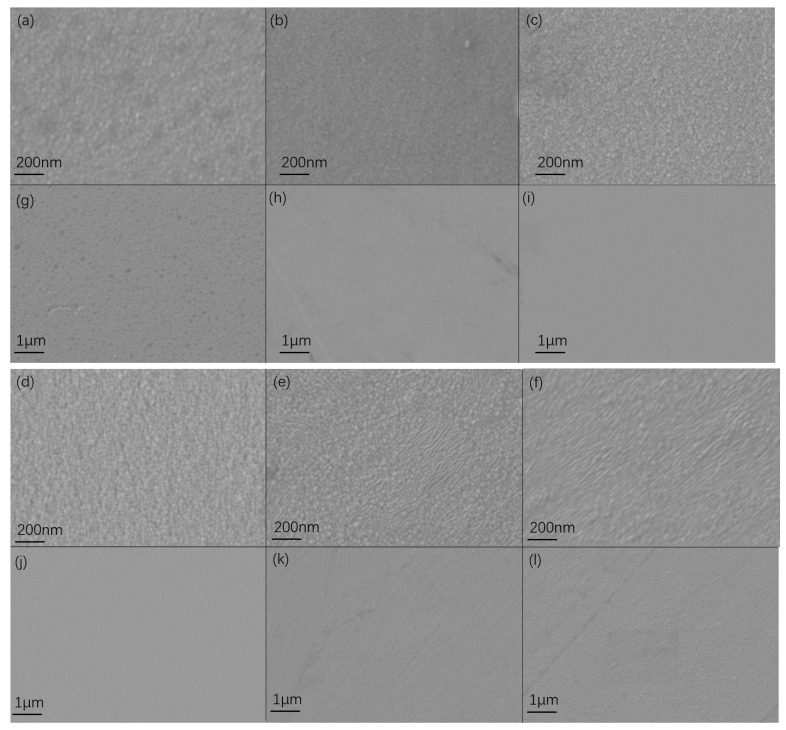
(**a**) Surface microstructure of Cu film prepared by DCMS; (**b**) surface microstructure of Cu film prepared by 30 μs and 1000 Hz; (**c**) surface microstructure of Cu film prepared by 50 μs and 600 Hz; (**d**) surface microstructure of Cu film prepared by 100 μs and 300 Hz;(**e**) surface microstructure of Cu film prepared by 200 μs and 150 Hz; (**f**) surface microstructure of Cu film prepared by 300 μs and 100 Hz; (**g**) surface microstructure of Cu film prepared by DCMS; (**h**) surface microstructure of Cu film prepared by 30 μs and 1000 Hz; (**i**) surface microstructure of Cu film prepared by 50 μs and 600 Hz; (**j**) surface microstructure of Cu film prepared by 100 μs and 300 Hz; (**k**) surface microstructure of Cu film prepared by 200 μs and 150 Hz; (**l**) surface microstructure of Cu film prepared by 300 μs and 100 Hz.

The surface of Cu films prepared with different parameters by SEM are shown in Figure 7. The surface of Cu film prepared by DCMS shows small particles and uneven pits on the surface. The Cu film prepared with a pulse width of 30 μs and 50 μs has an overall low actual power and low ion quantity, which means low energy, and the surface morphology presents small particles. When compared to the Cu film prepared by DCMS, the overall surface is smoother and there are no uneven pits. As the pulse width increases, the target material is fully discharged, and the overall actual power increases. When the pulse width is 100 μs, the microstructure of the Cu film surface grows. As the pulse width continues to grow, the particles with microscopic morphology on the surface of the Cu film begin to merge. At a pulse width of 200 μs, the microscopic surface morphology of the Cu film begins to exhibit elongated shapes. At a pulse width of 300 μs, the phenomenon of merging micromorphology on the surface of the Cu film intensifies and the micro-surface morphology of the Cu film begins to exhibit a branched morphology.

**Figure 8:**
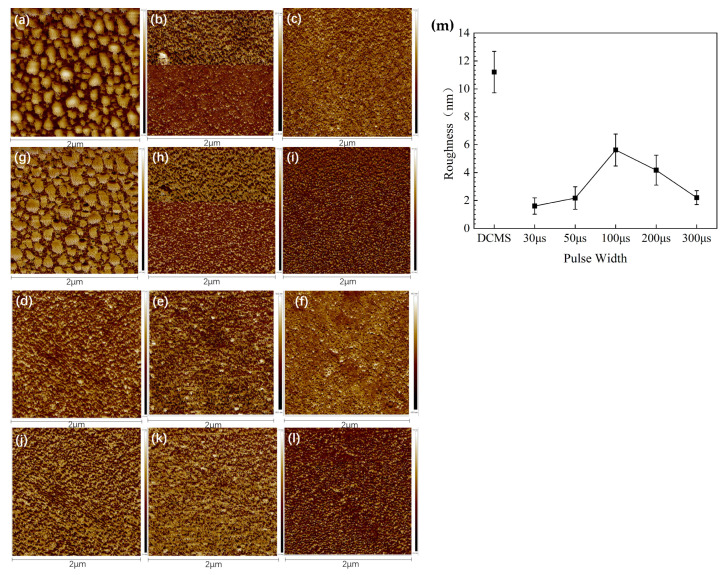
(**a**) AFM height measurement of DCMS; (**b**) AFM height measurement of 30 μs; (**c**) AFM height measurement of 50 μs; (**d**) AFM height measurement of 100 μs; (**e**) AFM height measurement of 200 μs; (**f**) AFM height measurement of 300 μs; (**g**) AFM phase measurement of DCMS; (**h**) AFM phase measurement of 30 μs S; (**i**) AFM phase measurement of 50 μs; (**j**) AFM phase measurement of 100 μs; (**k**) AFM phase measurement of 200 μs; (**l**) AFM phase measurement of 300 μs; (**m**) the roughness of Cu film prepared with different parameters.

The AFM height and phase measurements of Cu films prepared with different parameters is shown in Figure 8. The micro-surface of Cu prepared by DCMS and the micro-surface of Cu film prepared by HiPIMS exhibit significant differences. The micro-surface morphology of Cu film prepared by DCMS shows a hill-like shape, with overall unevenness. The surface of Cu film prepared by HiPIMS is smooth and even overall. 

The surface roughness of Cu film prepared by DCMS is much higher than that of copper film prepared by HiPIMS. The surface roughness of copper films prepared with different HiPIMS parameters is relatively similar. The overall surface roughness increases first and then decreases with the increase in pulse width. When the pulse width is 30 μs or 50 μs, due to the short pulse width, the surface microstructure of the Cu film obtained is small, resulting in a lower surface roughness of the Cu film. As the pulse width increases and reaches 100 μs, the surface microstructure and grain size grow, and the surface roughness of the Cu film increases. As the pulse width continues to increase, reaching 200 μs and above, the surface microstructure merges, merging from granular microstructure to elongated or branched microstructure, resulting in a further decrease in surface roughness.

### 3.3. Conductivity and Adhesion

Research has shown that, when copper is in the micron or below grain size range, the grain size significantly affects the electrical resistivity of copper, and the electrical resistivity of copper shows an inverse relationship with the grain size [37,38]. The presence of tensile stress can cause the atomic spacing between lattices to become farther, which is not conducive to the movement of electrons [39,40]. A microstructure that is too small will bring more interfaces, which will hinder the movement of electrons and cause a decrease in material conductivity and an increase in electrical resistivity. The results indicate that a too-short pulse width can lead to lower ion quantity and lower energy. Lower energy can make it difficult for the micro-surface morphology of the Cu film to obtain sufficient energy to grow, resulting in a smaller micro-surface morphology. Extending the pulse width of sputtering is beneficial for the growth and merging of the micro-surface morphology of Cu films. The growth and merging of micro-surface morphology can reduce the presence of interfaces in the material, improve the density of Cu film, and facilitate the growth of grains in Cu film, thereby reducing the resistivity of Cu film.

**Figure 9:**
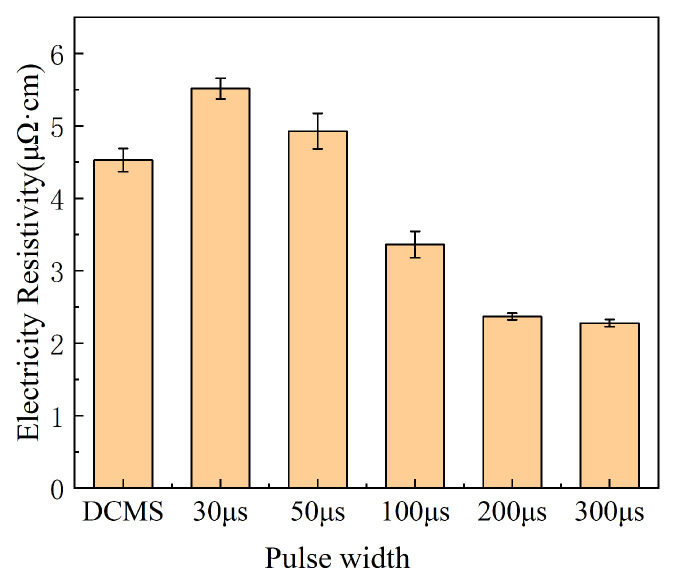
Resistivity Cu film prepared with different parameters.

The conductivity of copper films prepared with different parameters is shown in Figure 9. Due to insufficient discharge of the target material at pulse widths of 30 μs and 50 μs, the low ion quantity and actual power during sputtering result in insufficient growth energy for the thin film. The resistivity of the thin film is relatively high, and when the pulse width is 30 μs, the resistivity is 5.51 μΩ·cm. When the pulse width is 50 μs, the resistivity is 4.92 μΩ·cm, even lower than the copper film prepared by DCMS at the same power. The copper film prepared by DCMS has a resistivity of 4.53 μΩ·cm. As the pulse width prolongs, sputtering begins to enter the SS stage, and the ion quantity during sputtering increases, while the resistivity of the thin film begins to decrease. When the pulse width is 100 μs, the film resistivity is 3.36 μΩ·cm. When the pulse width is 200 μs, the film resistivity is 2.37 μΩ·cm. When the pulse width is 300 μs, the film resistivity is 2.27 μΩ·cm. When the pulse width is bigger than 200 μs, the resistivity of the thin film is already very close to the intrinsic resistivity of copper, which is 1.67 μΩ·cm [41]. Obviously, the difference in grain size between copper films prepared with long pulse width and those prepared with short pulse width is one of the reasons for the difference in their electrical resistivity. The presence of significant tensile stress is also one of the reasons why copper films prepared with short pulse widths have higher resistivity. The growth and merging of the micro-surface morphology of Cu films is also the reason why different pulse widths result in such a large difference in electrical resistivity.

**Figure 10:**
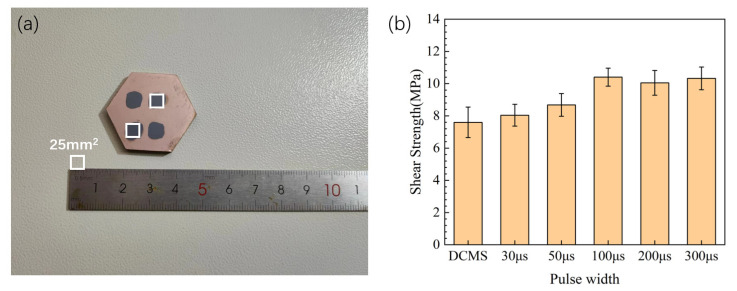
(**a**) Sampel after shear test; (**b**) shear strength of copper films prepared with different parameters.

The bonding strength of copper films prepared with different parameters on silicon carbide ceramics is shown in Figure 10b. The adhesive area of the metal block used in the shear peel test was 25 mm^2^ (5 mm × 5 mm). To ensure that the glue was applied evenly and does not escape during application, before applying the glue, a baffle with a 5 mm × 5 mm opening was placed on the test sample, the glue was applied to the baffle opening and a metal block was sticked. The sample after the shearing test is shown in Figure 10a.

The measurement method used in this study measures the shear strength between the copper film and the substrate. Due to the pulse discharge method of HiPIMS, the target material can achieve higher peak power density on same average power compared to DCMS. Therefore, the overall shear strength of copper films prepared by HiPIMS is higher than that of copper films prepared by DCMS. High sputtering energy can increase the injection depth of sputtered particles on the substrate, thereby enhancing the bonding strength, while low sputtering energy can lead to a decrease in bonding strength. When the pulse width is less than 100 μs, the actual power and ion quantity of the sputtered particles is low, resulting in a lower shear strength of the copper film. When the pulse width is 100 μs or greater, the sputtered particles obtain a higher ion quantity, resulting in an increase in the shear strength of the copper film.

## 4. Conclusions

**Figure 11:**
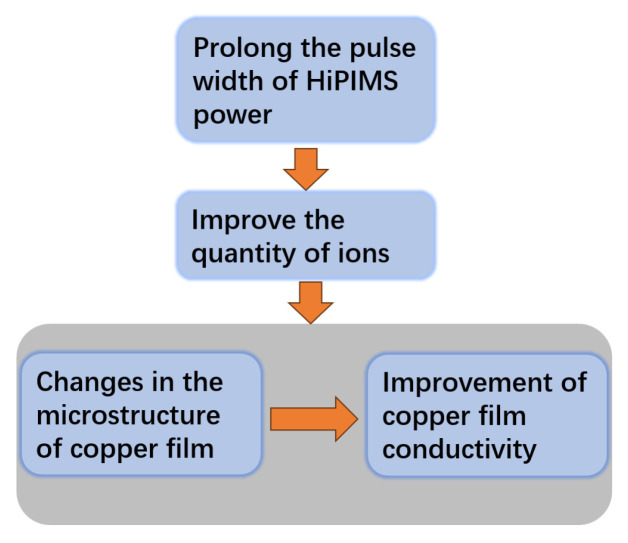
The graphical summary of the study.

In summary, this study investigated the effects of HiPIMS power supply parameters with different pulse times on the performance and structure of prepared Cu films, then compared them with copper films prepared by DCMS. The graphical summary of the study is shown in the Figure 11. We characterized the structure and properties of the thin film using a four-probe resistance tester, SEM, XRD, etc., and investigated the plasma state using a four-channel spectrometer. The film resistivity has reached 2.27 μΩ·cm and the film substrate bonding force reached about 10 MPa. Due to the high peak power density and pulse discharge characteristics of HiPIMS, the Cu film prepared by HiPIMS is superior to DCMS in terms of conductivity, bonding strength, and micro-surface roughness. Under the same sputtering voltage and duty cycle, a short pulse width of HiPIMS can lead to low ion quantity, resulting in poor performance of the prepared thin film. Extending the sputtering pulse width can enable sputtering to enter the SS stage, thereby improving the ion quantity. Extending the sputtering pulse width to 200 μs or more, the current waveform observed on the oscilloscope decreases from the first peak to a plateau period, indicating that sputtering has entered the SS stage. The ion quantity increases. This can promote the grain growth of Cu film, promote the merging of micro morphology on the surface of Cu film, reduce the micro interface of Cu film, facilitate the movement of electrons, improve the conductivity of the film, and reduce the surface roughness of the film. Therefore, extending the pulse width to a certain value can enter the SS stage of sputtering, increase the ion quantity during sputtering, and thus obtain higher quality Cu films. This provides new guidance for the industrial preparation of high-quality Cu films, and so, when utilizing HiPIMS to prepare copper films in the semiconductor industry, selecting an appropriate pulse width can effectively improve the conductivity of the copper film.

## Figures and Tables

**Table 1:** The sample parameters.

	Voltage (V)	Pulse Width (μs)	Duty Cycle	Average Power (W)	Temperature (°C)	Deposition Time (min)	Distance (mm)
1	535	30	3%	180	23	45	60
2	535	50	3%	180	23	45	60
3	535	100	3%	180	23	45	60
4	535	200	3%	180	23	45	60
5	535	300	3%	180	23	45	60
DCMS	370	-	100%	180	23	23	60

## Data Availability

Data are contained within the article.
